# Effects of Oxygen Flow during Fabrication by Magnetron Sputtering on Structure and Performance of Zr-Doped HfO_2_ Thin Films

**DOI:** 10.3390/ma16165559

**Published:** 2023-08-10

**Authors:** Yingxue Xi, Lei Liu, Jiwu Zhao, Xinhui Qin, Jin Zhang, Changming Zhang, Weiguo Liu

**Affiliations:** School of Optoelectronic Engineering, Xi’an Technological University, Xi’an 710021, China; liulei12271227@163.com (L.L.); zhaojiwu@st.xatu.edu.cn (J.Z.); 18792801300@163.com (X.Q.); j.zhang@xatu.edu.cn (J.Z.); zhangcm1995@163.com (C.Z.); wgliu@163.com (W.L.)

**Keywords:** magnetron sputtering, hafnium oxide-based heterojunction, ferroelectric thin film, oxygen mass flux, Zr-doped

## Abstract

Oxygen defects in Hafnium Oxide (HfO_2_)-based ferroelectric thin films not only are related to the cause of ferroelectricity but also affect the ferroelectric properties of the thin films. This paper, therefore, focuses on the fabrication of Zr:HfO_2_ thin films by RF (Radio Frequency) magnetron sputtering with Zr-doped HfO_2_ as the target and examines how oxygen flow impacts the oxygen vacancies and electrical properties thereof. Additionally, TiN thin-film electrodes were prepared by direct current (DC) magnetron reactive sputtering using nitrogen as the reaction gas, the influences of the substrate temperature on the film deposition rate and crystal phase structure were investigated, and the resultant thin-film electrodes with the lowest resistivity were obtained. Furthermore, the ferroelectric hysteresis loop and leakage current density of metal–insulator–metal (MIM) ferroelectric capacitors formed by annealing the 30 nm thick deposited Zr:HfO_2_ sandwiched between the top and bottom TiN electrodes were measured. The results demonstrate that varying oxygen flow has a considerable effect on oxygen vacancies and the Zr doping concentration of deposited Zr:HfO_2_ ferroelectric thin films. When the oxygen flow is set to 40 sccm (standard cubic centimeters per minute) and an external electric field strength of 2 mV/cm is applied, the remnant polarization reaches 18 μC/cm^2^, with a decrease in the leakage current density of 10^5–6^ orders of magnitude.

## 1. Introduction

Due to its low optical loss, high refractive index, high laser damage threshold, and good chemical and mechanical properties, Hafnium Oxide (HfO_2_) has become a major alternative material for the fabrication of optoelectronic thin films [1,2,3]. Featuring a high dielectric constant, wide band gap, low electron tunneling effect, and excellent CMOS (Complementary Metal–Oxide Semiconductor) compatibility, HfO_2_ can substitute for silicon oxide as the next-generation oxide gate dielectric material, hence finding wide applications in various memory devices, such as airplanes, medical electronics, smart meters, or cell phone chips [4,5,6]. In 2011, Müller et al. [7] revealed dielectric anomalies in doped HfO_2_ thin films, verifying their impressive ferroelectric properties. Given that HfO_2_ ferroelectric thin films can surmount the key problem of compatibility between traditional ferroelectric materials and the CMOS process and have the potential for miniaturization to enhance integration and storage capacity, doped HfO_2_ thin films should be highly promising for non-volatile memory (NVM) applications [8].

HfO_2_ thin films can be fabricated by Atomic Layer Deposition (ALD) [9], Physical Vapor Deposition (PVD) [10,11,12,13], Chemical Vapor Deposition (CVD) [14], or Pulsed Laser Deposition (PLD) [15]. Compared to other fabrication techniques, PVD-derived HfO_2_ thin films offer higher purity and superior performance [16,17], which can be achieved through the adjustment of parameters such as working pressure, sputtering power, gas flow ratio, and substrate temperature. Furthermore, Physical Vapor Deposition (PVD) techniques, such as magnetron sputtering, find wide use in industrial production for their superior automation and high output [18].

Doped HfO_2_ thin films can modify their lattice orientation, thus favoring the formation of an orthogonal phase featuring ferroelectricity. To date, laboratory experiments have managed to prepare ferroelectric HfO_2_ thin films doped with Zr [8], Y [17,19], Si [20], Al [21], and La [22]. Both ZrO_2_ and HfO_2_ are highly sought-after materials with similar physicochemical properties employed in the making of advanced metal–oxide–semiconductor field-effect transistors (MOSFETs) and high-K materials. In this regard, Mueller et al. [23] first reported the ferroelectric (FE) properties of Hf_0.5_Zr_0.5_O_2_ deposited by ALD. Moreover, Hf1-xZrxO_2_ materials drew wide attention in that they could crystallize into films at lower temperatures ranging from 400 to 600 °C [24], with the remnant polarization, dielectric constant, and content of the monoclinic phase in the films modulated by integrated components. The ferroelectric properties of Hf1-xZrxO_2_ thin films deposited by ALD were limited by the performance of such films, revealing a maximum thickness of 20 nm [25]. Magnetron sputtering, a coating technique widely used in industry, allows precise control over film components to achieve industrial production, although it is difficult to control the thickness at the atomic level. Oxygen defects in HfO_2_-based ferroelectric thin films bear relevance to the cause of ferroelectricity [26,27,28] and affect their ferroelectric properties as well. Zhou et al. [29] revealed that HfO_2_ ferroelectricity and wake-up and fatigue effects could be attributed to oxygen vacancies through first-principles calculations and that oxygen vacancies could stabilize the ferroelectric Polarization-Orthogonal (PO) phase through numerous experiments. Mitmann et al. [26] discovered that oxygen vacancies, when at an appropriate concentration, can positively impact the ferroelectric properties of HZO thin films. Baumgarten et al. [28] further confirmed the influences of oxygen flow on the number of oxygen vacancies in HfO_2_ thin films prepared through magnetron sputtering. In this paper, Zr-doped HfO_2_ thin films were fabricated by Radio Frequency (RF) magnetron sputtering by using a single zirconium-doped hafnium metal target under constant working air pressure, and the impact of oxygen flow on the film components was analyzed. A three-layer heterojunction consisting of TiN/X-HfO_2_/TiN was deposited onto a Si substrate, with TiN thin films deposited via DC magnetron sputtering as the electrode; subsequently, the components and electrical properties of the annealed HZO were examined.

## 2. Film Preparation and Characterization

**Figure 1:**
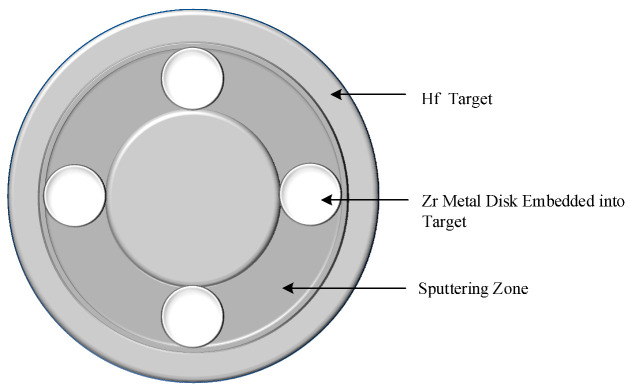
Schematic of Zr-doped hafnium magnetron sputtering cathode.

Doped HfO_2_ thin films were fabricated via RF magnetron sputtering on a P-type (100) single-crystal silicon substrate. The substrate was subjected to an ultrasonic cleaning procedure using acetone and anhydrous ethanol solution for 20 min prior to being coated, followed by rinsing with deionized water and blow-drying with nitrogen. In the fabrication of doped HfO_2_-based thin films by magnetron sputtering, a double-target co-sputtering approach is typically adopted [17,30,31,32,33]. As shown in Figure 1, a Zr-doped Hf target was employed in this study.

A Hf target with a size of Φ 60 mm × 5 mm and 99.99% purity was used as the magnetron sputtering cathode. Symmetrical circular holes of Φ 10 mm × 3 mm were cut at the magnetic field of the racetrack on the cathode target surface, where circular plates of Φ 10 mm × 3 mm made of doped Zr with purity ≥ 99.9% were uniformly installed. By using this method, uniformly doped HfO_2_ thin films were obtained. Moreover, the doping concentration could be modulated by controlling the number of circular element-doped plates in the magnetic field of the racetrack. The element-doped concentration could be characterized and calculated in line with the measurements of X-ray photoelectron spectroscopy (XPS). In this study, Thermo Scientific K-Alpha XPS (Waltham, MA, USA) was employed to analyze the components of the samples.

The coating process was conducted under conditions wherein the background vacuum was 2.0 × 10^−3^ Pa, with high-purity argon (≥99.99%) as the working gas and a target-to-substrate distance of 60 mm. In addition, prior to the coating process, the working pressure was set to 1 Pa, and a pre-sputtering process was performed for 15 min. Then, the coating experiment started. In fabricating TiN electrodes, pure nitrogen was introduced as the reaction gas, with an argon-flow-to-nitrogen-flow ratio of 10:1, wherein the argon flow was 30 sccm (standard cubic centimeter per minute, sccm), while the nitrogen flow was 3 sccm; the sputtering power was set to 140 W, and the working vacuum remained at 0.5 Pa by regulating the insert valve. The film thickness was regulated by appropriately adjusting the coating time to 20 min; this ensured an electrode thickness range between 30 nm and 40 nm. The resistivity of TiN films was calculated as a product of the thickness and sheet resistance measured by a four-point probe method. During the process of preparing doped HfO_2_ thin films, the target-to-substrate distance remained unchanged, with the sputtering power set to 100 W and the argon flow rate at 40 sccm; pure oxygen (≥99.99%) was introduced as the reaction gas, and the working vacuum was adjusted to 0.7 Pa without heating the substrate.

**Figure 2:**
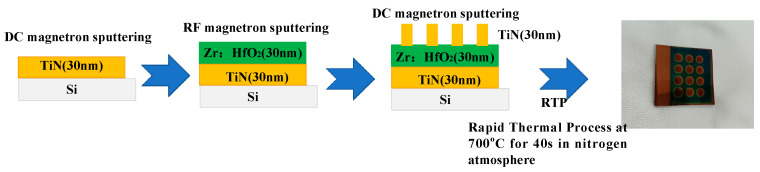
Fabrication flow of TiN/HZO/TiN/Si capacitor structure and photograph (see above right) of capacitor. The capacitor is fabricated through the deposition of Zr:HfO_2_ on a P-type silicon substrate by RF magnetron sputtering (top and bottom electrodes are TiN thin films deposited by DC magnetron sputtering), followed by annealing in a nitrogen atmosphere.

To investigate the electrical properties of doped HfO_2_ thin films, TiN electrodes were required to be coated on the top and bottom of the thin films. The preparation process and final samples are illustrated in Figure 2.

By using this method, a TiN/HfO_2_/TiN/Si heterojunction was formed through the deposition of a bottom TiN electrode, doped HfO_2_, and a top TiN electrode on a single-crystal silicon substrate. According to the competitive growth mechanism, the conductivity of TiN thin films is related to their lattice orientation, which can be controlled by tweaking the substrate temperature during the coating process.

**Figure 3:**
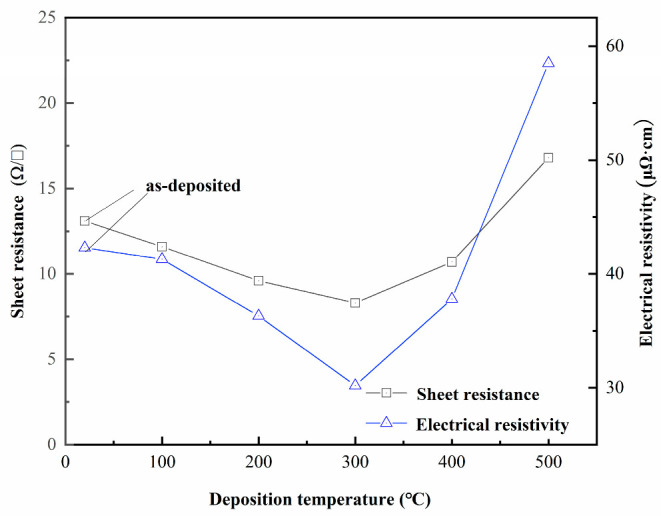
Sheet resistance (measured) and electrical resistivity (calculated) of TiN thin films deposited using magnetron sputtering at various substrate temperatures.

Figure 3 displays the sheet resistance and resistivity of TiN thin films deposited by DC magnetron sputtering at various substrate temperatures (100 °C, 200 °C, 300 °C, 400 °C, and 500 °C), with the latter obtained from the product of sheet resistance and film thickness.

**Figure 4:**
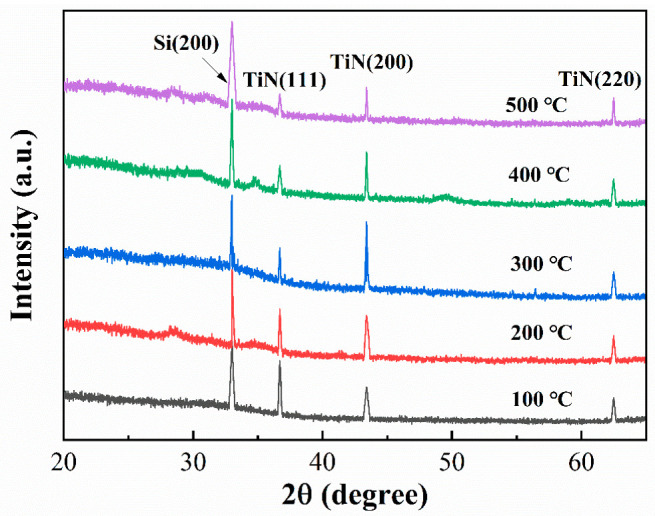
XRD patterns of TiN thin films deposited using magnetron sputtering at various substrate temperatures.

As the substrate temperature varied from 100 °C to 500 °C, the film thickness was found to be 35.6 nm, 37.8 nm, 36.4 nm, 35.3 nm, and 34.8 nm, accordingly. As illustrated in the figure, the sheet resistance and resistivity of the TiN thin film drop and then rise with the substrate temperature. At a substrate temperature of 300 °C, the sheet resistance is 8.3 Ω/□, which corresponds to a film resistivity of 30.2 µΩ·cm, slightly higher than that of the TiN block. This trend in the resistivity of TiN thin films may be attributable to their crystal structure. Figure 4 illustrates the grazing incidence scanning spectra of TiN thin films deposited at varying substrate temperatures with the Bruker D8 grazing incidence X-ray diffractometer.

Specifically, CuKα was used as the X-ray source, with a scan range from 10° to 80° and a grazing incidence angle of 1° at a scan rate of 2°/min. The analysis of the figure reveals that the preferred orientation of the crystal phase of TiN thin films transitions from (111) at 100 °C to (200) at 300 °C with increasing substrate temperature. As the substrate temperature continues to rise, each crystal phase of the thin films can obtain sufficient energy for competitive growth, eventually resulting in the polymorphism of the thin films. It has been established that thin films growing along (200) possess lower resistivity [34]. Thus, 300 °C was selected as the substrate temperature to fabricate TiN electrodes in this study.

At room temperature, the crystal phase of crystalline HfO_2_ is monoclinic [33], whereas high-temperature annealing is known to stimulate its transition from a monoclinic phase to a tetragonal phase, with its subsequent stabilization in a cubic phase. When HfO_2_ thin films are doped with other cations, the number of monoclinic phases in the films declines while that of tetragonal phases begins to rise, which indicates that the presence of tetragonal phases ultimately leads to the ferroelectricity of HfO_2_ thin films. The samples in this study were annealed in an RTP 500 rapid furnace for 40 s in a nitrogen atmosphere at 700 °C, with the heat treatment applied at a ramp-up rate of 35 °C/s, followed by a cooldown conducted in the furnace.

## 3. Results and Discussion

### 3.1. Variations in Deposition Rate and Components of Zr-Doped HfO_2_ Thin Films at Various Oxygen Flow Rates

**Figure 5:**
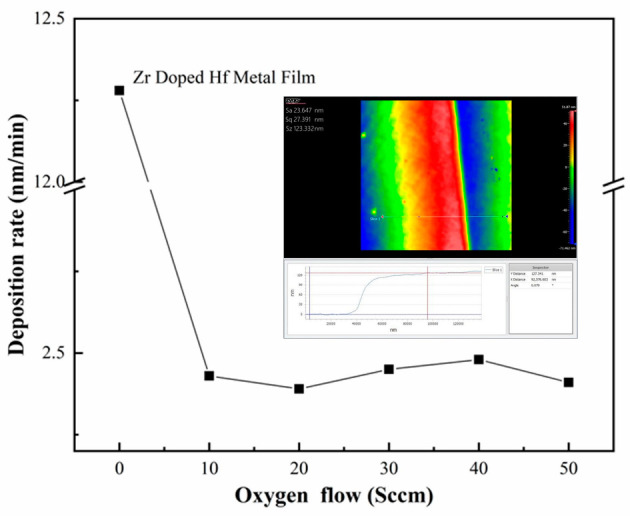
Graph of deposition rate of Zr-doped HfO_2_ thin films as function of oxygen flow rate ranging from 10 to 50 sccm. The thickness of Zr-doped HfO_2_ films prepared at an oxygen flow of 40 sccm was measured with a white-light interferometer (see the inset).

As illustrated in Figure 5, the variation curve of the deposition rate of HZO at various oxygen flow rates was obtained from the Zr-doped HfO_2_ thin film (HZO) experiment using RF magnetron sputtering, wherein the oxygen flow was set to 10 sccm, 20 sccm, 30 sccm, 40 sccm, and 50 sccm, with other process and conditions remaining the same.

Specifically, the inset shows the film thicknesses measured with a non-contact 3D surface profiler from ZYGO when film steps were obtained by masking at an oxygen flow rate of 40 sccm.

As shown in Figure 5, the deposition rate of thin films does not fluctuate with the rise in oxygen flow but remains unchanged when the oxygen flow is increased from 10 sccm to 50 sccm in a given process range with a fixed working air pressure. Among all the elements that affect the deposition rate, the target power, working air pressure, and oxygen flow take the top three places in terms of importance [35]. In principle, at a lower oxygen flow rate, thin films are deposited in the metallic mode at an accelerated pace. As shown in the figure, the deposition rate of the doped metal film reaches 12.8 nm/min in the oxygen-free mode, which is far greater than that of the film in the reactive mode in the oxygen condition. However, an excessively high concentration of oxygen leads to target poisoning, subsequently resulting in a lower deposition rate. In this paper, it is evident that the deposition rate remained unaffected by alterations in oxygen flow, suggesting that the conditions chosen for the deposition of the thin films were well suited.

**Figure 6:**
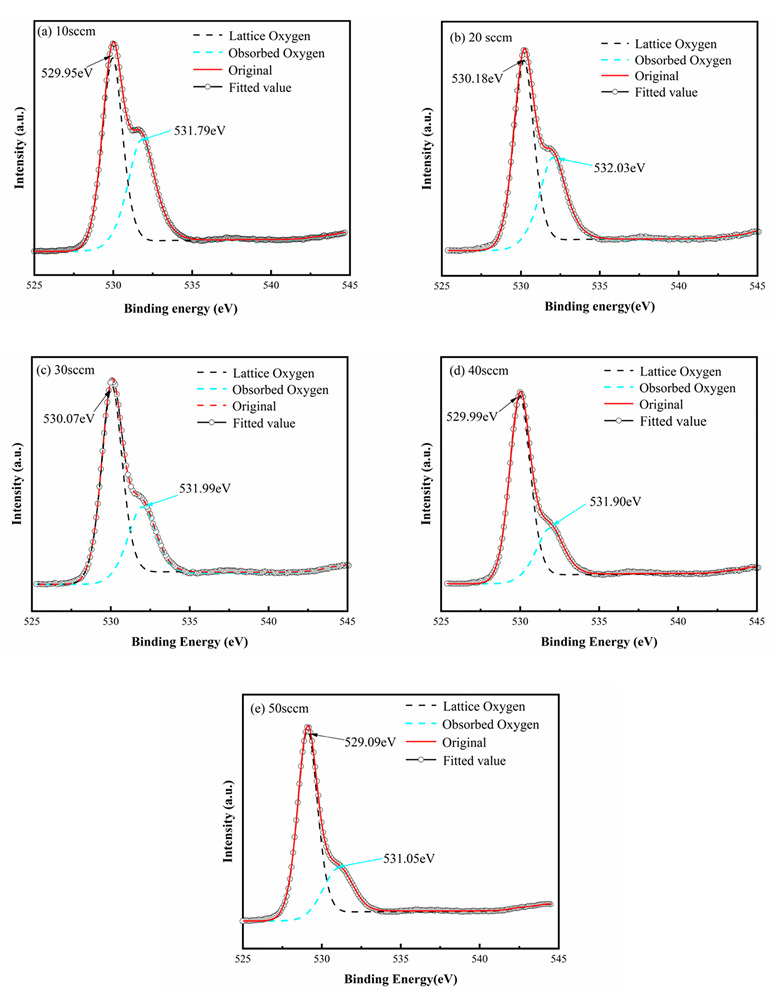
XPS O1s fine spectra of Zr-doped HfO_2_ thin films deposited at various oxygen flow rates.

The Zr-doped HfO_2_ thin films obtained at various oxygen flow rates were tested with X-ray photoelectron spectroscopy (XPS) after a 40 s Ar^+^-etching procedure was performed on the surfaces of the films to remove contaminants on them. Subsequently, XPS results were analyzed using the Avantage software, and the O1s fine spectrum fitting graphs of the Zr-doped HfO_2_ thin films were successfully fitted, as shown in Figure 6.

Figure 6a presents the O1s fine spectrum fitting graphs of the thin films deposited at an oxygen flow rate of 10 sccm. The main peak of the binding energy, 529.37 eV, falling within the O1s (529.00~530.00 eV) binding energy range of metal oxide compounds, corresponds to Zr-O and Hf-O bonds [36], whereas the minor peak at 531.63 eV corresponds to the oxygen vacancies present within the films [17,37]. Figure 6b–e illustrate the fitting results of O1s fine spectra obtained at oxygen flow rates of 20 sccm, 30 sccm, 40 sccm, and 50 sccm, respectively. It can be observed that the main peaks characterizing Zr-O and Hf-O bonds and minor peaks characterizing oxygen vacancies arise in all spectra. The minor peak area is reduced correspondingly as the oxygen flow increases, suggesting that oxygen vacancies drop in number with increasing oxygen flow.

**Figure 7:**
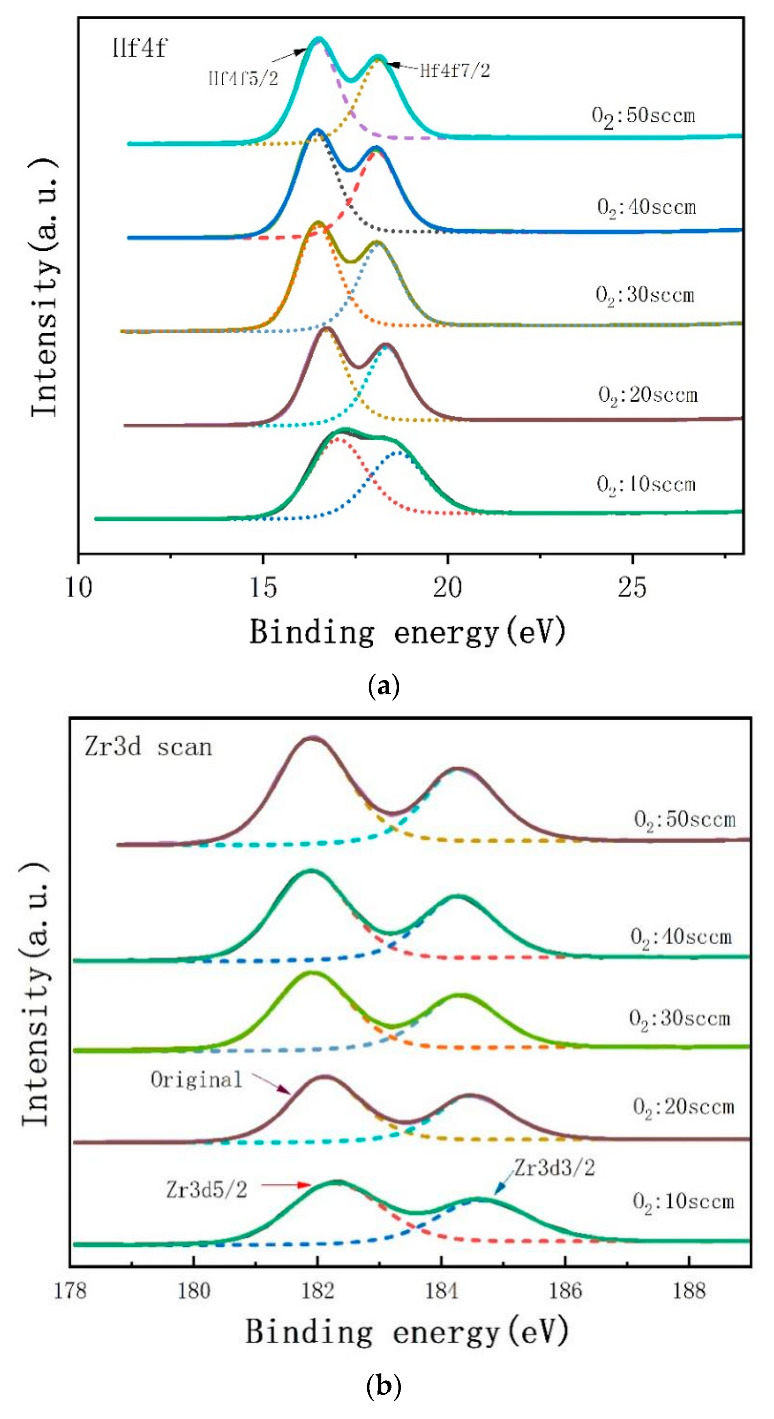
XPS fine spectra of Hf-4f (**a**) and Zr-3d (**b**) of Zr-doped HfO_2_ thin films deposited at varying oxygen flow rates.

Figure 7 illustrates the fine scanning spectra of photoelectron spectroscopy of Hf-4f and Zr-3d peaks of Zr-doped HfO_2_ thin films deposited at varying oxygen flow rates.

By means of Gaussian Peak Fitting of Hf-4f, the spin–orbit-splitting-induced Hf-4f7/2 and Hf-4f5/2 peaks are identified, respectively, at 16.1 eV and 17.8 eV. The splitting energy is 1.7 eV, which is in agreement with the findings reported in the literature that characterizes the Hf-O bond in HfO_2_ thin films [37], implying that Hf exists in the form of Hf^+4^. Although the binding energy of the Hf 4f peak of HfO*_x_*_<2_ has not yet been reported, it is plausible that the binding energy of the Hf-4f peak shifts closer to the Hf atom as oxygen defects in the films grow. In contrast, the HfO_2_ spectrum measured in the figure is characterized by a bimodal binding energy of Hf-4f, which shifts toward the Hf peak as the oxygen flow increases. This is because the air pressure remained constant during the preparation. Consequently, as the oxygen flow rises, a concomitant rise in the vacuum pumping rate is required to maintain constant air pressure. A plausible explanation is that neutral oxygen vacancies formed during the process of film deposition are almost completely replaced by two electrons from the Hf atom [38]. This novel double-site occupancy defect state would promote a shift in the Fermi energy level [39], thereby leading to the augmentation of the binding energy of the Hf-4f peak. Further experiments and theoretical investigations are necessary to expound on this phenomenon. According to the figure, increased oxygen flow caused a progressive reduction in oxygen vacancies in the films and, consequently, resulted in a steady fall in the binding energy of the Hf-4f peak. However, when the oxygen flow rate was 50 sccm, the defects and vacancies in the deposited films increased, resulting in the Hf-4f peak binding energy no longer declining. In fact, research [29,40] has confirmed that oxygen vacancies are the primary driver of the ferroelectric phase of Hf-based thin films in a metastable state, and thus, the effective regulation of these vacancies in thin films is essential in forming the ferroelectric phase.

A similar phenomenon to the Hf-4f peak was also observed in the Zr-3d peak, where the binding energy was affected by the transfer of nearby O_2_ charges. As depicted in Figure 7b, the Zr-3d peak splits into two peaks corresponding to spin–orbit-splitting-induced Zr-3d5/2 and Zr-3d3/2 at 184.62 eV and 184.62 eV [41], respectively, implying the presence of Zr-O bonds in the films. As illustrated in Figure 7b, the bonding energy of the two peaks of Zr-3d declines with increasing oxygen flow below 40 sccm, yet it marginally rises at the oxygen flow rate of 50 sccm. By taking account of the ratio of the peak area of Hf-4f to that of Zr-3d, the content of Zr can be calculated at the different oxygen flow rates as follows: 12 mol.%, 14 mol.%, 15 mol.%, 15.3 mol.%, and 18 mol.%. It can be seen that both the Hf-4f peak and Zr-3d peak shift toward a lower binding energy position when the oxygen flow is augmented. This implies that oxygen vacancies within the films decrease with the increase in oxygen flow, and the difference in their shift magnitudes signifies that some of the Hf-O bonds are substituted by Zr-O bonds.

**Figure 8:**
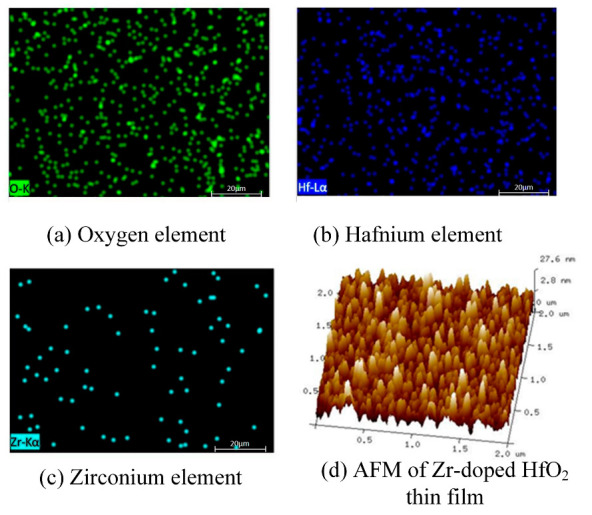
EDS spectra and AFM of Zr-doped HfO_2_ thin films deposited at an oxygen flow of 40 sccm. (**a**) Oxygen element, (**b**) hafnium element, (**c**) zirconium element (**d**) AFM.

Figure 8 shows the X-ray energy dispersion spectrum and AFM pattern of Zr-HfO_2_ thin films prepared at an oxygen flow rate of 40 sccm. As shown in the figure, the Hf, O_2,_ and Zr elements in the film are randomly and uniformly distributed without segregation, which proves that the elemental distribution of the film deposited by reactive sputtering using a zirconium-doped hafnium target is uniform. The AFM pattern shows that the deposited film is uniform, and the grain size is between 10 and 20 nm. Root-mean-square (RMS) surface roughness is 7.03 nm.

### 3.2. HZO-Based Heterojunction I–V Test and Polarization Curve Measurement

**Figure 9:**
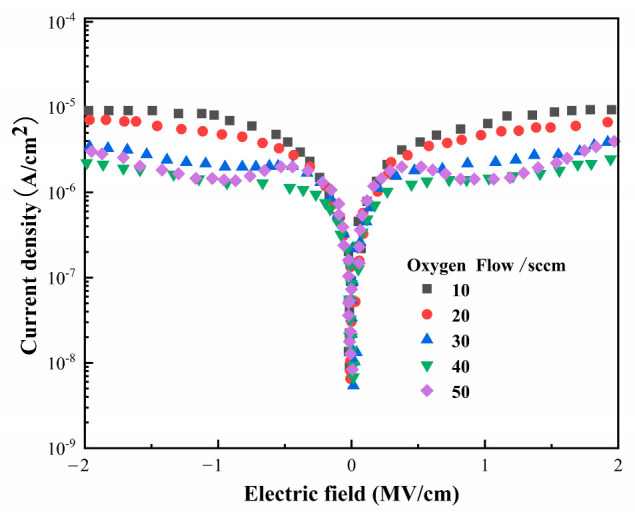
I–V curves of Zr-doped HfO_2_ thin films deposited at different oxygen flow rates.

Figure 9 shows the I–V characteristic curves of the TiN/HZO/TiN heterojunction fabricated at various oxygen flow rates. It is evident from the figure that the heterojunction features a low leakage current. Such a feature can help reduce the loss of the device and improve its reliability and durability [42]. The asymmetry of the leakage current curves is due to the electrostatic potential barriers generated between the TiN electrode material and HZO thin film, and the presence of oxygen vacancies gives the curves a bulged shape. An analysis of the I-E curve reveals that, when an external electric field of 2 MV/cm is applied, the leakage current in the heterojunction decreases gradually as the oxygen flow increases from 10 sccm to 40 sccm; at an oxygen flow rate of 40 sccm, a minimum leakage current of approximately 10^−5.6^ A/cm^2^ is achieved; however, an increase in the oxygen flow to 50 sccm results in a further increase in the leakage current. When the oxygen flow rises, the vacuum pumping rate increases automatically to maintain the stable working pressure, leading to an increase in oxygen partial pressure and a drop in argon partial pressure at the working pressure. Eventually, the deposition rate of thin films declines slightly at an oxygen flow rate of 50 sccm (as exhibited in Figure 5). However, an unwanted increase in oxygen can lead to more defects in the films and, thus, a higher energy leakage in capacitors.

**Figure 10:**
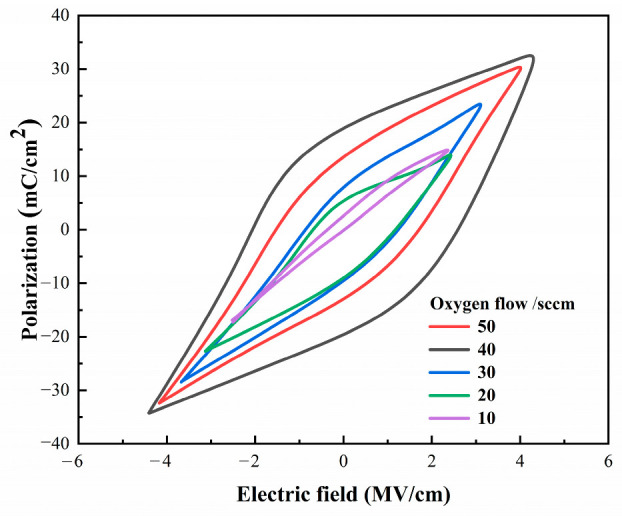
P–E curves of Zr-doped HfO_2_ thin films deposited at various oxygen flow rates.

Using a ferroelectric analyzer, a polarization-intensity–external electric field (P-E) test was conducted on a Zr-doped HfO_2_-based heterojunction. Figure 10 displays the results of the P-E ferroelectric hysteresis loop.

At an oxygen flow rate of 10 sccm, the capacitor exhibits a linear orientation instead of a hysteresis curve, represented as paraelectricity. As the oxygen flow increases, the capacitor demonstrates obvious ferroelectricity characteristics, reaching a maximum remnant polarization of approximately 18 µC/cm^2^ and a coercive electric field of 2 MV/cm when the oxygen flow gradually increases to 40 sccm. However, with the oxygen flow increasing to 50 sccm, both the remnant polarization intensity and coercive electric field begin to decrease, possibly resulting from the excessive oxygen flow, which may have led to an increment in defects in the fabricated Zr-doped HfO_2_ thin films. In this study, Zr:HfO_2_ thin films showed good ferroelectricity and a maximum Zr doping amount of up to 20% mol. Moreover, its ferroelectric properties correlated with oxygen vacancies. Compared to the thin films fabricated by Atomic Layer Deposition (ALD), the films prepared herein remained unaffected by size effects, with a thickness of up to 30 nm. Additionally, the Zr:HfO_2_ heterojunction possessed an enhanced remnant polarization intensity and band gap, making it thermally stable and compatible with Complementary Metal–Oxide Semiconductors (CMOSs) and thus capable of being fabricated by 3D integration technology.

## 4. Conclusions

In this study, a Zr-doped TiN/HfO_2_/TiN heterojunction was fabricated on the Si substrate by RF magnetron sputtering. The study focused on the analysis of the chemical components and oxygen vacancies in Zr:HfO_2_ thin films prepared at varying oxygen flow rates and their impacts on the ferroelectric characteristics of the heterojunction. According to XPS analysis results, an increase in oxygen flow led to a diminishing number of oxygen vacancies in the films, and these vacancies were regarded as what caused the ferroelectricity of the films. Conversely, when the oxygen flow reached 50 sccm, a surplus of oxygen led to the heightening of defects in films. This result had a considerable effect on the electrical properties of the films, wherein the leakage current of the Zr-doped HfO_2_-based heterojunction fluctuated as the oxygen flow increased from 10 sccm to 50 sccm, with the small remnant polarization getting larger and then smaller. At the appropriate oxygen flow rate of 40 sccm, the remnant polarization intensity (Pr) attained a maximum of 18 µC/cm^2^, with the coercive electric field (Ec) amounting to approximately 2 MV/cm and the Zr-doped thin films reaching a thickness of 30 nm.

## Data Availability

Not applicable.
